# High-Efficiency and Low-Damage Lapping Process Optimization

**DOI:** 10.3390/ma13030569

**Published:** 2020-01-24

**Authors:** Ci Song, Feng Shi, Wanli Zhang, Zhifan Lin, Yuxuan Lin

**Affiliations:** Laboratory of Science and Technology on Integrated Logistics Support, College of Intelligence Science and Technology, National University of Defense Technology, Changsha 410073, China; shifeng@nudt.edu.cn (F.S.); linzhifan16@nudt.edu.cn (Z.L.); zhangwanli17@nudt.edu.cn (W.Z.); zhongdayuu@gmail.com (Y.L.)

**Keywords:** low-damage fabrication, effective removal rate of damage, lapping process, sub-surface damage

## Abstract

The silica opticsare widely applied in the modern laser system, and its fabrication is always the research focus. In the manufacturing process, the lapping process occurs between grinding and final polishing. However, lapping processes optimizations focus on decreasing the depth of sub-surface damage (SSD) or improving lapping efficiency individually. So, the optimum balance point between efficiency and damageshould be studied further. This manuscript establishes the effective removal rate of damage (ERRD)model, and the relationship between the ERRD and processing parameters is simulated. Then, high-efficiency, low-damage lapping processing routine is established based on the simulation. The correctness and feasibility are validated. In this work, the optimized method is confirmed that it can improve efficiency and decrease damage layer depth in the lapping process which promotes the development of optics in low-damage fabrication.

## 1. Introduction

Fused silica optics with excellent surface and sub-surface qualityare widely used in high power laser system [1]. For example, the system of National Ignition Facility (NIF) contains thousands of fused silica optics that precisely guide, reflect, amplify, and focus 192 laser beams onto a fusion target [2]. However, the laser induced-damage initiation on fused silica optics limits the performance of high-power laser applications [3]. In the previous study, some researchers have shown that sub-surface damage (SSD)is likely to be laser damage precursors [4]. It can cause laser damage susceptibility regionally, enhance laser absorption, and then introduce macroscopic damage to optics [5,6]. In order to avoid degradation of quality, SSD must be minimized or eliminated in the fabrication of optics.

**Figure 1:**
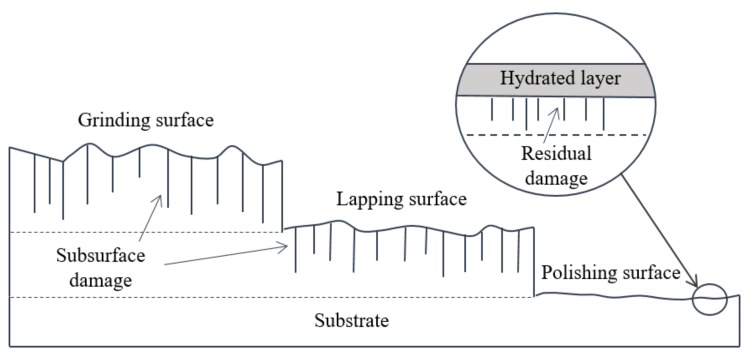
The schematic view of sub-surface damage (SSD) from grinding to polishing.

The optical fabrication process mainly contains grinding, lapping and polishing.SSD is introduced in the grinding process and restrained in the following process [7,8,9]. Figure 1 illustrates the generation of SSD during manufacturing. To remove SSD in grinding processing, it needs to remove materials to 3~9 times of grinding abrasive size in the lapping process, which is time-consuming and inefficient [10]. For the lapping process, the material removal occurs into two types: brittle domain and plastic domain. The division of material removal domain is related to an external load and the properties of the material itself. In the brittle domain, cracks appear on the surface and materials are sheared off from substrate under the load, which can achieve higher material removal efficiency. Differently, materials are removed through the function of plastic flow in the plastic domain whose removal is relatively inefficient. In the previous study, a high-efficiency lapping process tends to increase the depth of SSD and delay the processing cycle since the material removal rate of final polishing is rather low. On the other hand, if processing efficiency is reduced to control SSD, more time would be consumed [11]. The main problem is that efficiency and SSD controlling are not balanced in the lapping process.

Buijs et al. had found that the energy required for material fracture removal was lower than that for plastic deformation, and the critical load of median cracks of fused silica was 0.02N through Vickersindentation experiment. A model was also established to predicted removal efficiency [12]. Lambropoulos et al. had proposed an interpretation of manufacturing featuresbased on the optical material removal micromechanical model, which could conduct the fabrication process [13]. More recently, Neauport et al. studied the effect of lapping parameters with different lapping slurries on roughness and SSD. After lapping under the optimal parameters (abrasive: Al_2_O_3_, grain size: 3 μm, lapping speed: 50 rpm, load: 2.8 kg), theroughness R_t_of fused silica is 1.3 μm and SSD depth is 4.3 μm, while material removal rate was relatively small [14]. From above all the researches, they focused ondecreasing the depth of SSD or improving lapping efficiency individually. 

To optimize the lapping process, a theoretical model is proposed and optimal lapping parameters are applied in the manufacture of optics. This work is organized as follows: Section 1 is Introduction. Section 2 presentsthe model and basic equation, which describes the establishment of the remove rate model, subsurface defect model and the effective removal rate of damage (ERRD) model in detail. Section 3 is the simulation while Section 4 is the experimental validation. Section 5 is the conclusion of the work. In general, the theoretical model and the optimal lapping parameters can be a reference on high-efficiency, low-damage optics fabrication.

## 2. Model and Basic Equation

### 2.1. Remove Rate Model and Subsurface Defect Model

**Figure 2:**
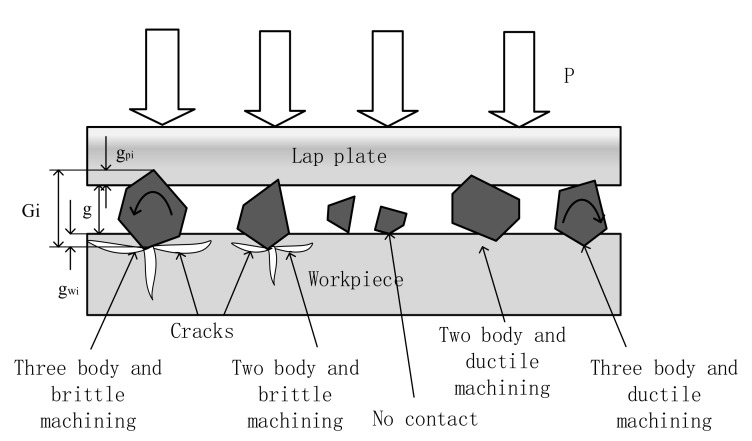
Interaction among abrasive, lapping plate and workpiece.

In reality, as shown in Figure 2, the material is often removed by a combination of various mechanical mechanisms [15]. This co-existence of mechanisms is believed to be caused by non-uniform depth of penetrations among abrasives. Usually, abrasives with large sizes would embed in both the workpiece surface and lapping plate, bear the load, and then remove the materials from the workpiece along withthe motion of the lapping plate. Conversely, abrasives with small size adhere to the surface, which can not achieve effective material removal.It should be noted that cracks appeared along with material removal (brittle domain) when an external load is large enough. Meanwhile, smaller hardness of lapping plate or workpiece would promote the function of rolling of abrasives and finally achieves three-body machining. Considering the hardness of iron lapping plate and external load, the model focuses on three-body and brittle machining [11].

In the three-body and brittle lapping process, the force borne by the single abrasive can be defined as in Equation (1) [15,16].

(1) $$F_{i}=\alpha \cdot {\left(x-g\right)}^{2}/{\left(1/\sqrt{H_{w}}+1/\sqrt{H_{p}}\right)}^{2},$$

where *α* is geometric constant depends on the abrasive shape, it can be calculated as *α*; = 4∙tan^2^ψ, ψ is the sharpness angle of abrasive; *x* is the size of abrasive; *g* is the gap between workpiece and lapping plate; *H*_w_ is the hardness of workpiece; *H*_p_ is the hardness of lapping plate.

The total load F can be expressed as Equation (2).

(2) $$\left\{\begin{array}{l} F=\sum_{x_{i}\;>\;g}F_{i}=\;N\int_{g}^{x_{\max}}\left[F\left(x\right)\cdot \varphi \left(x\right)\right]dx=\frac{\alpha \cdot N}{{\left(1/\sqrt{H_{w}}+1/\sqrt{H_{p}}\right)}^{2}}\cdot \int_{g}^{x_{\max}}\left[{\left(x-g\right)}^{2}\cdot \varphi \left(x\right)\right]dx\ldots \ldots 1)\\ \varphi \left(x\right)=\frac{f(x)}{\int_{x\min}^{x\max}f(x)dx}=\frac{e^{\frac{1}{2}\frac{\ln(x-a_{1})a_{2}{}^{2}}{a_{3}}}}{\sqrt{2}(x-a_{1})a_{3}\int_{x\min}^{x\max}f(x)dx}\ldots \ldots 2) \end{array}\right.,$$

where *φ*(*x*) is bounded lognormal distribution; *N* is the total number of abrasive, it can be calculated by Equation (3) [17].

(3) $$\left\{\begin{array}{l} N=\frac{A\cdot x_{\max}}{V\cdot (1+\frac{\rho_{a}}{m\rho_{l}})}\ldots \ldots 1)\\ V={\frac{\pi \cdot (x_{\mathrm{avg}})}{6}}^{3}\ldots \ldots 2) \end{array}\right.,$$

where A is the contact area between lapping plate and workpiece; *ρ*_a_ and *ρ*_l_ is the density of abrasive particles and lapping slurry respectively; *m* is the weight ratio of abrasive particles in lapping slurry.

**Figure 3:**
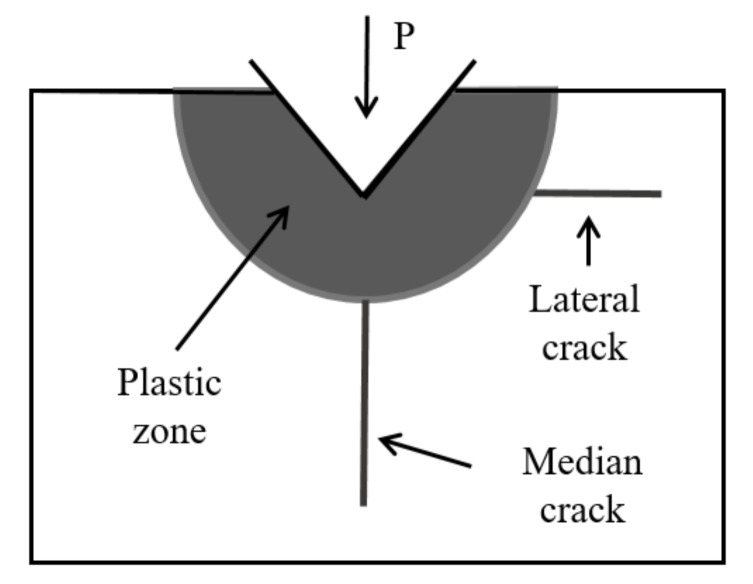
Micro-indentation mechanics.

Some researchers have derived theoretical equations of median crack depth and lateral crack depth, based on indentation of sharp indenter [18]. Figure 3 illustrates the crack systems under indentation.

According to previous study, the depth of SSD is equal to that of median crack *Cm*, and it can be written as Equation (4).

(4) $$Cm=\alpha_{K}{}^{2/3}\cdot {\left(\frac{E_{w}}{H_{w}}\right)}^{(1-m)2/3}\cdot {\left(\cot\psi \right)}^{4/9}\cdot {\left(\frac{F}{K_{}}\right)}^{2/3},$$

where *E*_w_ is the elastic modulus; *H*_w_ is the hardness of material; *α*_k_ is a dimensionless constant, which can be defined as *α*_k_ = 0.027 + 0.090(*m*-1/3), the value of *m* is 0.5; *F* is the load that lapping plate exerts; *K* is thedynamic fracture toughness related to the properties of materials.

The lateral crack is related to the removal rate, the length *CL* can be defined as Equation (5).

(5) $$CL={\left(\frac{\xi }{M}\right)}^{1/2}\cdot {\left(\cot\psi \right)}^{5/12}\cdot \frac{E_{w}{}^{3/8}}{K^{1/2}H_{\begin{array}{l} w \end{array}}{}^{1/2}}\cdot F^{5/8}$$

where *ξ* is a constant which can be obtained through experiment; *M* is a geometrical constant, *M* = 0.75.

The material removal rate (MRR) can be expressed as Equation (6).

(6) $$\left\{\begin{array}{l} MRR=n\frac{dv}{dt}\ldots \ldots 1)\\ \frac{dv}{dt}=\pi {\left(CL\right)}^{2}\cdot (Cm)\cdot \frac{2V}{\pi s_{avg}}\ldots \ldots 2)\\ s_{avg}=\frac{\int_{d}^{x\max}x\varphi (x)dx}{\int_{d}^{x\max}\varphi (x)dx}\ldots \ldots 3) \end{array}\right.,$$

where *V* is abrasive velocity; *n* is the effective number of abrasive, which can be written as:

(7) $$n=N\int_{g}^{x_{\max}}\varphi \left(x\right)dx.$$

### 2.2. ERRD Model

The optimization of efficiency and SSD are two important targets inthe lapping process. However, they cannot be achieved at the same time, because of lacking in theoretical analysis. To quantify the efficiency and SSD, the ERRD model isproposed. It can be expressed as follows:

(8) $$\mathrm{ERRD}=\frac{{\mathrm{SSD}}_{before}{-\mathrm{SSD}}_{after}}{t}$$

(9) $$t=\frac{H\times S_{workpiece}}{MRR}$$

(10) $$\mathrm{ERRD}=\frac{{(\mathrm{SSD}}_{before}{-\mathrm{SSD}}_{after})\times \mathrm{MRR}}{H\times S_{workpiece}},$$

where *K* is ERRD, its unit is μm/min; H is removal depth in lapping process, its unit is μm; t is processing time, its unit is min;S is the area of surface, its unit is mm^2^; MRR is the volume removal efficiency in lapping process, its unit is μm^3^/min.

It should be noted that SSD represents the length of the median crack. In order to simplify the calculation, we use the damage depth to replace it, and its unit is μm.

The ERRD model can be used to find the balance point between SSD and removal efficiency. According to Equations (8) and (9), the value of ERRD is positively related to material removal rate under the same condition of SSD. Moreover, it shows negative correlation between ERRD and removal rate under the same condition of removal efficiency, according to Equation (10). The optimization of SSD and removal efficiency could refer to the ERRD model.

## 3. Simulations

The factors affecting removal efficiency and SSD in the lapping process mainly include abrasive, pressure and lapping velocity [19]. To optimize efficiency and control the depth of SSD, the relationship between ERRD and these factors were analyzed.

(1) Abrasive

The type and particle size are two key factors thataffect the efficiency and depth of SSD [20]. In this work, different abrasive types and particle sizes are chosen to simulate.

**Figure 4:**
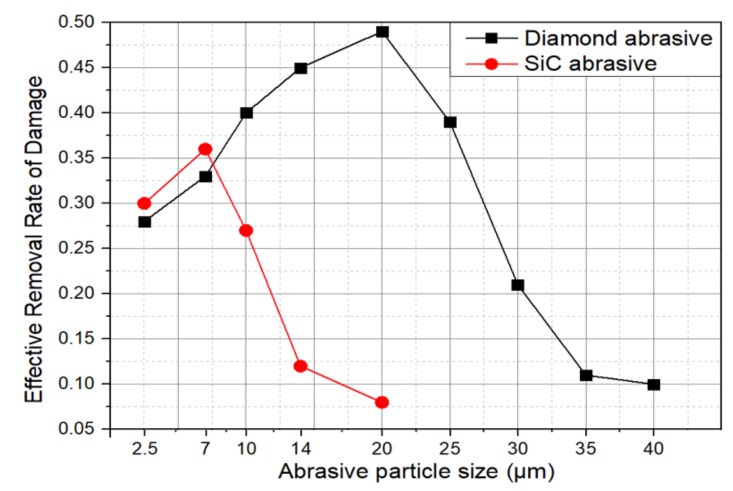
The relationship between effective removal rate of damage (ERRD) and abrasive.

The processing parameters are set as: 40 mm lapping tool size, 0.1 MPa lapping pressure, and 120 rpm lapping velocity. The relationship between ERRD and abrasive can be obtained from simulation, as shown in Figure 4.

Based on the results, it can be concluded that: (1) ERRD value of diamond abrasive is 1.5~3 times of silicon carbide (SiC)with the same particle size, except for W2.5 and W7. (2) Both curves of ERRD increase firstly and then reduce. (3) The largest ERRD value of silicon carbide (SiC)achieves when particle size is W7 (7 μm), while that of diamond is W20 (20 μm).

(2) Pressure

**Figure 5:**
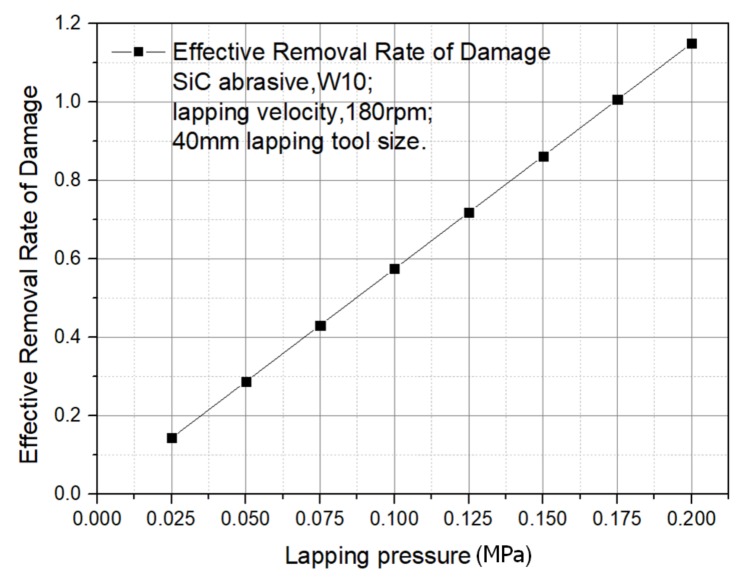
ERRD for various lapping pressure.

The processing parameters are set as:40 mm lapping tool size, silicon carbide abrasive, W10 abrasive, and 180 rpm lapping velocity.The relationship between ERRD and lapping pressure can be obtained from the results, as shown in Figure 5. It can be seen ERRD is linear with lapping pressure, which means that large pressure tends to improve ERRD under the conditions of stable process parameters.

(3) Velocity

The process parameters are 40 mm lapping tool size, silicon carbide abrasive, W10 abrasive, and 0.2 MPa lapping pressure.

**Figure 6:**
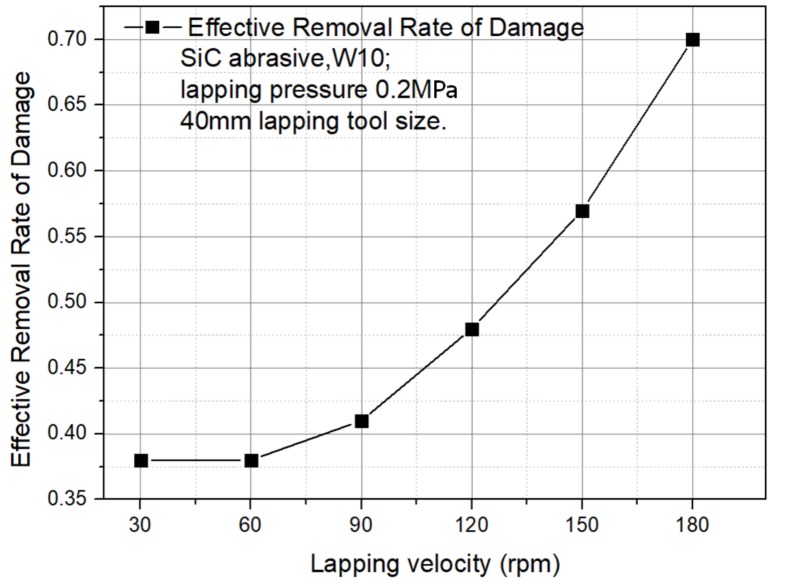
ERRD for different lapping velocity.

The relationship between the ERRD and lapping velocity can be obtained from Figure 6. According to the result, ERRD is nonlinear with lapping velocity. When lapping velocity beyond 90 rpm, the value of ERRD grows faster.

## 4. Experimental Validation

(1) Validation of simulation parameters

**Figure 7:**
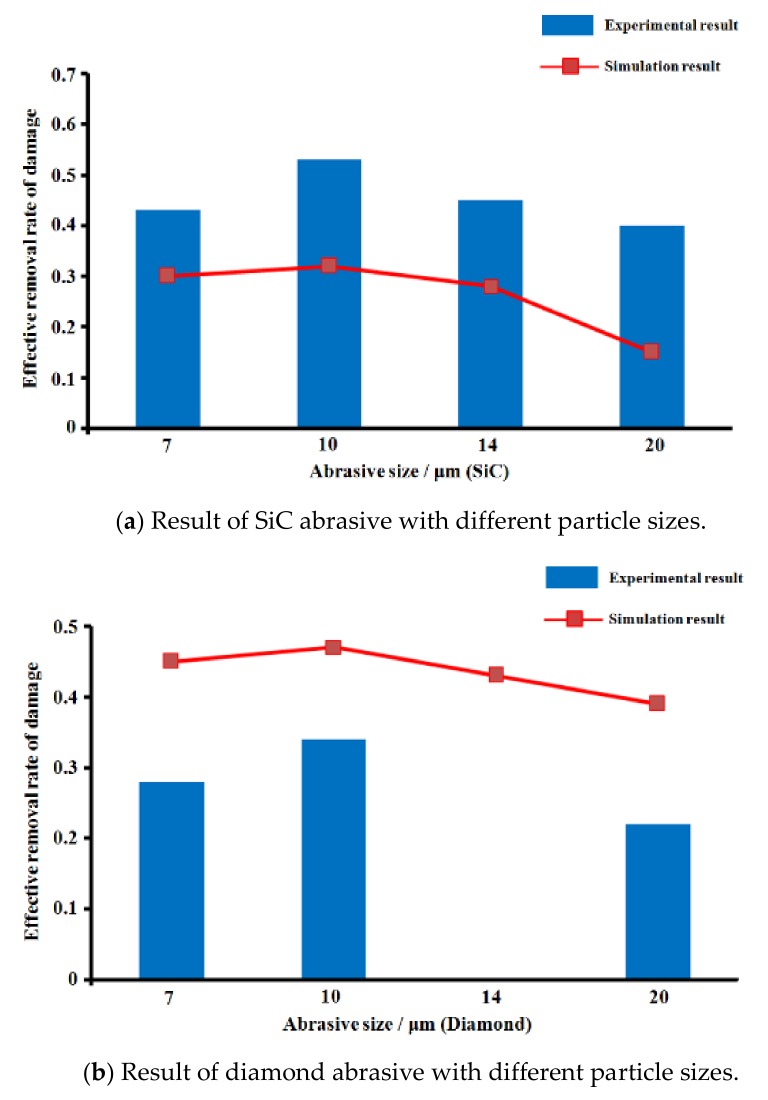
Results of ERRD with different abrasive.

After the ERRD model is established, we carried out a verification experiment to verify the accuracy of the model. In the verification experiment, abrasives with different particle sizeswereused as verified objects. Corining 7980 fused silica samples (size: 10 mm × 10 mm, number: 7) wereused as the materials, concentration of lapping slurry is 5%, lapping velocity is 120 rpm, 0.2 MPa lapping pressure, and 200 μm material removal depth. The results are shown in Figure 7.

**Figure 8:**
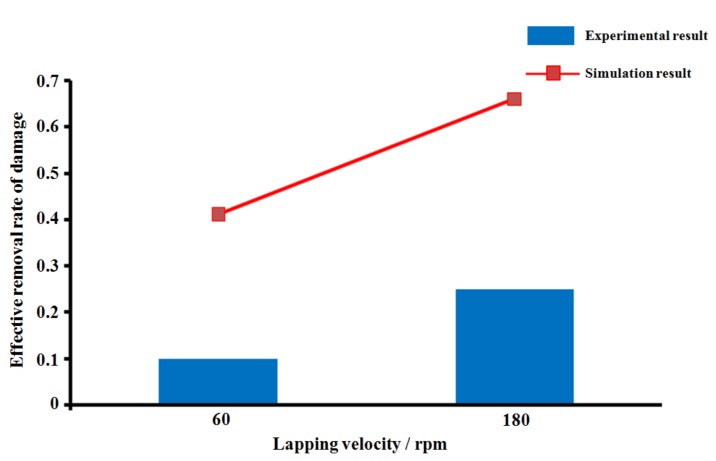
Result of ERRD under different lapping velocity.

According to the results shown in Figure 7, ERRD (effective removal rate of damage) of SiC abrasive washigher than that of diamond abrasive under the same conditions. In Figure 7a, the values of simulation results werelower than experimental results, whichwerecontrary to the results shown in Figure 7b. For Figure 7a, the highest ERRD occurredwhen 10 μmSiC abrasiveswereused, and its value was0.55 μm/min. For Figure 7b, the highest ERRD value was 0.33 μm/min, while the diamond abrasive size 10 μm. Though the experiment results weredifferent from the simulation, their trends werethe same. In consideration of ERRD, 10 μmSiC abrasives can be applied in the lapping process. (14 μm diamond abrasive is not applied in the experiment because of the limitation of resource in our team). Afterwards, the lapping velocity is regarded as the object and the result is shown in Figure 8.

In Figure 8, the trend of experimental results is the same as that of simulation results. According to Equation (10), a higher material removal rate (MRR) would improve the value of ERRD. Limited by the performance of the machine tool, the lapping velocity can be set at 180 rpm. It should be noted that the processing environment, conditions of lapping slurry and other factors may influence experimental results.

(2) Validation of rough and fine lapping process.

The simulation in Section 3 and Section 4.(1) provides references to optimize lapping parameters. In the actual lapping process, lapping pressure needs to be controlled strictly.Excessive pressure would push the lappingslurry out of the lapping area and make direct contact between the lappingplate and thesurface [21].Limited by machine tool, the velocity is not more than 180 rpm. According to Figure 4, W20 diamond abrasive and W7 SiC abrasive are utilized in the experiment. The parameters are all listed in Table 1. The total material removal amount in Process A and Process B are the same asthe conventional lapping process.

Three Corning 7980 fused silica sampleswith 100 mm × 100 mm (sample 1–4) wereprepared on a single side polishing machine. Sample 1waslapped by the conventional process.Sample 2 and sample 3werelapped by processesA and B, respectively. Sample 4 waslapped by a rough and fine lapping process. Then, four samples werepolished by the MRF wedge method, and the damage depth wasmeasured by the microscope.

**Figure 9:**
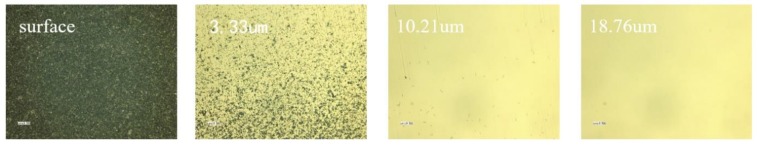
SSD results of sample 1.

As shown in Figure 9, cracks existed on the initial surface, and theiramount reduces when the polishing depth increases. The cracks almost disappear when the polishing depth increases to 18.76 μm. Therefore, the damage depth of sample 1 should be 18.76 μm.

**Figure 10:**
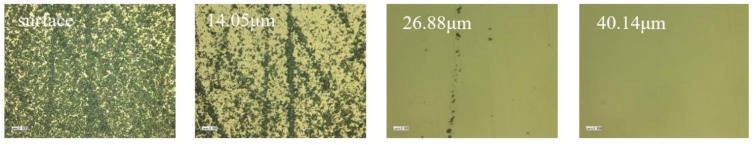
SSD results of sample 2.

**Figure 11:**
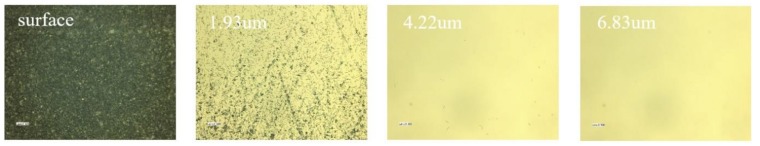
SSD results of sample 3.

The results of sample 2 show thatcracks reduce when depth increases, see Figure 10. However, it still exists at the depth of 26.88 μm where cracksalready disappear on sample 1. Therefore, we keep on lapping, and the cracks disappear at the depth of 40.14 μm. As shown in Figure 11, it can be clearly seen that the damage depth of sample 3 is 6.83 μm. Compared with the conventional lapping process, process B can significantly decrease the depth of SSD.

The detailed results listed in Table 2 are to evaluate the effect under different lapping parameters. We can find that the efficiency of process A is the highest among the three processes, while the SSD depth of process B is the shallowest.

**Figure 12:**
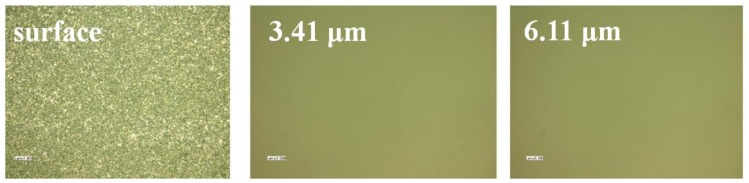
SSD results of sample 4.

As shown in Table 3, we choose W20 diamond and W7 SiCas the rough lapping abrasive and fine lapping abrasive, respectively.Theremoval amount is 200 μm in rough lapping and 50 μm in fine lapping. The lapping pressure is 0.2 MPa and velocity is 180 rpm. Total lapping time is 3.1 h (2h rough lapping and 1.1 h fine lapping)and the lapping results are shown in Figure 12.

In Figure 12, the SSD depth of the sample is 6.11 μm after the lapping process. Compared with the conventional lapping process, the combination of rough and fine lapping processes was more efficient, which reduced lapping timeby 65% with the same material removal (250 μm).Meanwhile, the SSD depth reduces from 18.76 μm to 6.11 μm. The results demonstrated that rough and fine lapping can not only reduce SSD depth but also improve the removal efficiency.

## 5. Conclusions

In this work, the ERRD model is established to provide a theoretical reference about balancing lapping efficiency and SSD, the relationship between ERRD, abrasive type, abrasive size, pressure and velocity is simulated. Through the simulation, W7 SiC abrasive and W20 diamond abrasive can be applied in the lapping process, while lapping pressure needs to be controlled strictly. Limited by the performance of the machine tool, the lapping velocity is set at 180 rpm. Based on the simulation, the validation experiments are carried out. In the experiment, the optimal parameters are applied to rough lapping and fine lapping process. After lapping, the depth of SSD decreases from 18.76 μm to 6.11 μm and processing time decreased from 9 h to 3.1 h, reduces by 65% compared with conventional parameters. The results indicate that the optimum lapping process can not only improve efficiency but also reduce SSD depth. Therefore, the ERRD model and optimum lapping process can be applied in high-efficiency, low-damage lapping process optimization for high power laser optics.

## Figures and Tables

**Table 1:** Process parameters in the lapping process.

Item	Conventional Lapping	Process A	Process B
Material removal amount	250 μm	250 μm	250 μm
Abrasive	SiC	Diamond	SiC
Abrasive size	W20	W20	W7
Pressure	0.2 MPa	0.2 MPa	0.2 MPa
Velocity	120 rpm	180 rpm	180 rpm

**Table 2:** Results under different lapping parameters.

Item	Conventional Process	Process A	Process B
Lapping Time (h)	9	2.5	5.3
SSD depth (μm)	18.76	40.14	6.83

**Table 3:** Parameter of optimum lapping process.

Item	Rough Lapping	Fine Lapping
Material removal amount	200 μm	50 μm
Abrasive	W20 Diamond	W7 SiC
Pressure	0.2 MPa	0.2 MPa
Velocity	180 rpm	180 rpm
